# Oxidative Stability of Phytosterols in Camellia Seed Oil During Heating: The Impact of Different Antioxidants

**DOI:** 10.3390/foods14132297

**Published:** 2025-06-28

**Authors:** Dongkun Zhao, Xin Wang, Sicong You, Lijuan Wang, Usman Amjad, Baocheng Xu, Xinjing Dou, Lili Liu

**Affiliations:** 1 College of Food and Bioengineering, Henan University of Science and Technology Henan International Joint Laboratory of Food Green Processing and Safety Control, Luoyang 471000, China; kun18238796961@163.com (D.Z.); wangxin_327@163.com (X.W.); yousicong@126.com (S.Y.); usmanamjad293@gmail.com (U.A.); douxj521@163.com (X.D.); yangliuyilang@126.com (L.L.); 2 College of Basic Medical Science, Ningxia Medical University, Yinchuan 750004, China; mnn717@163.com; 3 National Experimental Teaching Demonstration Center of Food Processing and Safety, Henan University of Science and Technology, Luoyang 471000, China

**Keywords:** phytosterols, camellia seed oil, heating process, antioxidant, phytosterol oxidation products

## Abstract

Phytosterols (PS) have specific oxidation rules in different lipid media. After oxidation, PS will form oxidation products, which has potential physiological toxicity to the human body. Camellia seed oil (CSO) is a unique emerging edible oil in China. This oil has a fatty acid composition similar to olive oil, in which oleic acid is dominant. In order to solve the thermal oxidation of PS in CSO at high temperature (180 °C), we studied its antioxidant strategy by evaluating different antioxidants. Four antioxidants—BHA, TBHQ, epigallocatechin gallate (EGCG), and α-tocopherol (VE)—along with one synergist, citric acid (CA), were selected and used in this study. The antioxidant effects of different combinations (single antioxidant, single antioxidant + CA, mixed antioxidant, mixed antioxidant + CA) were compared. After 180 min of heating, the PS and phytosterols oxidation products (7α-hydroxy-, 7β-hydroxy-, 5α,6α-epoxy-, 5β,6β-epoxy-, 7-keto-, and trihydroxy-PS) were estimated by GC-MS. Through comparative analysis, the results showed that the combination of mixed antioxidants and CA had the best antioxidant effect, and the inhibition rate of VE + TBHQ +CA was as high as 42%, which had a breakthrough significance for stabilizing the thermal oxidation of PS in camellia seed oil. At the same time, it also provides a valuable reference for ensuring the edible safety of camellia seed oil in Chinese food heating habits.

## 1. Introduction

In China, camellia seed oil (CSO) is recognized as the “Oriental olive oil” due to its fatty acid profile closely resembling that of olive oil, with oleic acid content exceeding 68% [1,2]. Notably, CSO contains substantial amounts of bioactive compounds, including phytosterols (PS), tocopherols, and squalene [3], positioning it as a high-value edible oil with rapidly growing consumption in the Chinese market. PS, a group of structurally analogous phytochemicals, constitute the predominant component of the nonsaponifiable fraction in CSO [4,5]. These compounds exhibit multifunctional bioactivities, such as cholesterol-lowering effects [6,7,8], cardiovascular protection [9], and anti-carcinogenic/anti-inflammatory properties [10,11]. However, Chinese culinary practices frequently involve high-temperature cooking methods like frying and roasting, which accelerate PS oxidation compared to storage conditions [12]. This thermal processing leads to significant formation of phytosterol oxidation products (POPs) [13], including 7-hydroxy-, 7-keto-, epoxy-, and triol derivatives [14] associated with potential health risks [15,16]. Consequently, enhancing PS thermal stability and suppressing POPs formation has become an urgent priority given CSO’s expanding consumption.

The oxidation of PS in CSO during heating follows a free radical-mediated chain reaction mechanism. To address this, antioxidant intervention emerges as the most practical and cost-effective strategy. Primary antioxidants (e.g., phenolic compounds) terminate oxidation chains by scavenging free radicals, while secondary antioxidants (e.g., metal chelators) inhibit oxidation initiation through pro-oxidant neutralization. Notably, developing effective antioxidant strategies for PS in CSO requires comprehensive consideration of multiple factors: (1) fatty acid composition and their positional distribution in triacylglycerol (TAG) molecular architecture, (2) antioxidant chemical structures and concentrations, and (3) system environmental conditions. Particular attention should be paid to fatty acid profiles, given their complex interactions with PS oxidation. While Zhao et al. [17] observed that unsaturated lipid matrices inhibited POPs formation (with protection efficiency increasing with unsaturation degree), contradictory findings emerged from subsequent studies. Xu et al. [18] demonstrated time-dependent effects: saturated/monounsaturated fatty acids (stearic/oleic acids) initially protected PS better than polyunsaturated counterparts (linoleic/α-linolenic acids) during the first 30 min at 180 °C but accelerated PS degradation after 60 min. This time-dependent behavior may stem from differential radical generation rates and oxygen competition mechanisms [19]. During the initial phase, polyunsaturated fatty acids (PUFAs) demonstrate higher susceptibility to free radical generation compared to their saturated counterparts. This heightened reactivity facilitates the initiation of self-propagating oxidation chain reactions, thereby accelerating PS degradation through radical-mediated processes. Subsequently, as the oxidation process progressed, two critical factors became apparent: (1) the gradual depletion of dissolved oxygen within the reaction microenvironment, and (2) the competitive oxygen consumption dynamics between PUFAs and PS. Under oxygen-limited conditions, the preferential oxidation of PUFAs establishes a molecular shielding mechanism, thereby conferring enhanced preservation of PS structural integrity in PUFA-containing systems when compared to saturated fatty acid matrices during extended oxidation periods. Given that CSO’s unique fatty acid profile (oleic acid: 68–77%; palmitic: 11–13%; linoleic: 7–14%; stearic: 2–4% [20]), differs markedly from common vegetable oils, its PS degradation kinetics and antioxidant requirements likely exhibit distinct characteristics [13,21,22]. However, systematic studies investigating the thermal oxidation of PS in CSO and corresponding antioxidant strategies have not been thoroughly explored. To address this gap, comprehensive experimental evaluations should be conducted to assess the efficacy of various antioxidant approaches in inhibiting PS oxidation, with the ultimate goal of developing a potentially applicable antioxidant protocol for PS during CSO thermal processing.

Antioxidants inhibit oxidation through distinct mechanisms with varying efficacy, broadly categorized as primary and secondary antioxidants based on their action modes. Primary antioxidants (e.g., phenolic compounds) neutralize free radicals via hydrogen atom donation, while secondary antioxidants (e.g., citric acid) mitigate metal ion pro-oxidant effects by forming thermodynamically stable complexes and reducing redox potentials. In this study, the selection of antioxidants was based on their distinct mechanisms and compatibility with edible oil systems. Butyl hydroxyanisole (BHA) and tertiary butylhydroquinone (TBHQ), as synthetic phenolic antioxidants, exhibit efficient radical-scavenging capabilities, making them suitable for inhibition of the thermal oxidation chain reaction of PS. α-Tocopherol (VE), a natural component of vegetable oils, acts as a chain-breaking antioxidant by donating hydrogen to lipid radicals. Epigallocatechin gallate (EGCG), a polyphenol from green tea, combines radical scavenging with metal-chelating properties to inhibit oxidation. Citric acid (CA) serves as a synergist by chelating pro-oxidant metal ions (e.g., Fe^2+^, Cu^2+^), reducing their catalytic activity in radical generation. This combination—comprising primary antioxidants (BHA, TBHQ, VE, EGCG) and a secondary antioxidant (CA)—targets multiple oxidation pathways, mimicking industrial antioxidant blends to provide comprehensive protection against PS oxidation in CSO during heating. The antioxidant activity of primary antioxidants is intrinsically linked to their chemical structure, which governs their reactivity toward free radicals. Zhang et al. [23] demonstrated that the free radical scavenging capacity (assessed via 2,2-diphenyl-1-picrylhydrazyl, DPPH assay) of common antioxidants follows this descending order: α-tocopherol > TBHQ > BHA > butylated hydroxytoluene (BHT). Notably, however, radical scavenging activity does not consistently correlate with anti-lipid-oxidation performance. For instance, in fish oil emulsions, the lipid oxidation inhibition efficacy of these antioxidants followed a distinct sequence: BHA > α-tocopherol > TBHQ > BHT [23]. This discrepancy may be attributed to substrate-specific interactions and the physical state of the system. To develop an effective antioxidant strategy for inhibiting PS degradation during CSO thermal processing, we selected four radical scavengers—BHA, TBHQ, EGCG, and VE—alongside CA as a metal chelator. The proposed synergistic mechanism operates as follows: CA sequesters metal ions in CSO through chelation, while the phenolic antioxidants collectively disrupt PS oxidation chain reactions. Specifically, BHA, TBHQ, VE, and EGCG may act complementarily to maintain the system’s reduced state via electron transfer, simultaneously regenerating primary antioxidants through hydrogen atom replenishment [24]. This integrated approach targets multiple oxidation pathways to enhance protection efficacy.

The present study aims to evaluate the efficacy of individual antioxidants and their synergistic combinations in stabilizing PS during CSO thermal processing. By monitoring peroxide value, PS degradation, and POPs formation under controlled heating, we developed a comprehensive stabilization protocol to mitigate PS oxidation. The optimized strategy demonstrates translational potential for practical food processing applications (e.g., roasting or frying), where its implementation could substantially reduce both the generation and dietary exposure to POPs.

## 2. Materials and Methods

### 2.1. Chemicals and Reagents

PS mixture (≥95% GC purity) was procured from Tianbao Biotechnology Co., Ltd. (Xi’an, China). The following analytical standards were obtained from Sigma-Aldrich (Shanghai, China): campesterol (≥98%), stigmasterol (≥95%), β-sitosterol (≥97%), cholestanol (analytical grade), 7α-hydroxycholesterol (≥95%), 7β-hydroxycholesterol (≥95%), 5α,6α-epoxycholesterol (≥95%), 5β,6β-epoxycholesterol (≥95%), cholestanetriol (≥98%), 7-ketocholesterol (≥90%), 19-hydroxycholesterol (analytical grade), N-methyl-N-(trimethylsilyl)heptafluorobutyramide (MSHFBA), and α-tocopherol (≥96%). Sinopharm Chemical Reagent Co., Ltd. (Shanghai, China) supplied: 1-methylimidazole (1-MIM, ≥99%), HPLC-grade *n*-hexane, anhydrous diethyl ether (freshly distilled prior to use), HPLC-grade acetone, HPLC-grade dichloromethane, potassium hydroxide, anhydrous ethanol, n-butanol, ferrous chloride, barium chloride, hydrochloric acid, and citric acid. Bailingwei Scientific (Beijing, China) provided BHA (≥99%), TBHQ, EGCG (≥98%), and sodium sulfate. Ultra-pure water was generated using a Milli-Q^®^ water purification system (Millipore Corporation, Burlington, MA, USA). Solid-phase extraction cartridges (ProElut™ NH2, 1.0 g/6 mL) were procured from Dikma Technologies Inc. (Beijing, China). All chemicals were of analytical grade or higher unless specifically noted.

### 2.2. Oil Sample

The Camellia oleifera seed oil used in this study was prepared in our laboratory. Fresh camellia seeds procured from local markets were dehydrated in a thermostatically controlled oven at 45 °C for 6 h prior to mechanical pressing. Oil extraction followed our established cold-pressing methodology as detailed in previous work [25]. The crude oil underwent sequential purification: dissolution in *n*-hexane followed by chromatographic filtration through activated carbon (deodorization), activated clay (decolorization), and combined neutral alumina/silica gel columns (phytosterol/tocopherol and polar impurity removal) [26,27]. Post-treatment, solvents were eliminated via rotary evaporation to yield purified CSO triglycerides, which were stored in amber glass vials at −20 °C under nitrogen atmosphere. The key physicochemical characteristics of the processed CSO included the following: (1) fatty acid profile—oleic acid (C18:1n-9c, 80.6%), linoleic acid (C18:2n-6c, 7.8%), palmitic acid (C16:0, 7.1%), stearic acid (C18:0, 3.1%), linolenic acid (C18:3n-3, 0.1%), myristic acid (C14:0, 0.4%), palmitoleic acid (C16:1n-7, 0.1%), arachidic acid (C20:0, 0.2%), eicosenoic acid (C20:1n-9, 0.3%), erucic acid (C22:1n-9, 0.2%), and tetracosenoic acid (C24:1 n-9, 0.1%). (2) The peroxide value was 0.4 meq O_2_/kg oil. (3) The acid value was <0.1 mg KOH/g oil (below method quantification limit).

### 2.3. Sample Preparation

A homogeneous PS-enriched camellia seed oil (CSO) system was prepared by dis-solving 50 mg PS mixture (0.5% *w/w*) and 9.950 g purified CSO in *n*-hexane within a 50 mL volumetric flask, followed by vortex mixing (2000 rpm, 3 min) to yield Sample T (200 mg/mL in *n*-hexane). Antioxidant stock solutions (1 mg/mL in acetone) were prepared for α-tocopherol, EGCG, TBHQ, BHA, and CA.

The experimental design comprised five treatment groups: (1) Single antioxidant treatment: 2 mL of Sample T (containing 400 mg PS-CSO) was supplemented with 80 μL antioxidant solution, achieving a final concentration of 0.02% (*w/w*) antioxidant in the PS + CSO mixture. (2) Antioxidant + synergist treatment: 400 mg of PS-CSO was supplemented with 80 μL antioxidant solution and 80 μL CA solution, each achieving 0.02% (*w/w*) in the final mixture. (3) Binary antioxidants treatment: 400 mg of PS-CSO was supplemented with 40 μL of Antioxidant A solution and 40 μL of Antioxidant B solution, each achieving 0.01% (*w/w*) in the final mixture. (4) Binary antioxidants + synergist treatment: 400 mg of PS-CSO was supplemented with 40 μL of Antioxidant A solution, 40 μL of Antioxidant B solution, and 80 μL CA solution, achieving final concentrations of 0.01% (*w/w*), 0.01% (*w/w*), and 0.02% (*w/w*) in the mixture, respectively. (5) Control: Antioxidant-free PS-CSO system. All treatments were conducted in standardized iron reactors (15 mm diameter × 85 mm height) under atmosphere conditions.

The prepared samples were first vortex-mixed at 2000 rpm for 3 min, followed by solvent evaporation under a gentle nitrogen stream. Subsequently, thermal treatment was conducted in a precision temperature-controlled oven (NDO-400, EYELA, Tokyo, Japan) at 180 °C for 3 h. After natural cooling to ambient temperature (25 ± 2 °C), the samples were redissolved with 2 mL of acetone and stored in light-protected containers at −20 °C. All analyses were required to be completed within 48 h of sample preparation.

### 2.4. Peroxide Value

The peroxide value (PV) was determined according to the method of Shantha and Decker [28] with modifications. Three stock solutions were prepared: (1) 3.94 M sodium thiocyanate (NaSCN) aqueous solution, (2) 32.75 mM barium chloride dihydrate in 0.2 M HCl, and (3) 35.97 mM ferrous sulfate heptahydrate (FeSO_4_·7H_2_O) in 0.2 M HCl. An Fe^2+^ working solution was prepared by mixing equal volumes of barium chloride and FeSO_4_ solutions. This mixture was then combined with an equal volume of 3.94 M NaSCN solution, followed by 100-fold dilution with 0.2 M HCl to yield Solution A.

For PV analysis, 10 μL of thermally oxidized sample (prepared as per Section 2.3) was dissolved in 190 μL of methanol/n-butanol (2:1, *v/v*) through vortex mixing (30 s, 2500 rpm) to obtain Solution B. Aliquots (10 μL) of Solution B were transferred to a 96-well microplate, mixed with 190 μL of Solution A using 10 cycles of aspiration/dispense mixing, and incubated in light-protected conditions at 25 °C for 20 min. Absorbance was measured at 510 nm using a microplate reader. Quantification was performed against a cumene hydroperoxide standard curve. Results, expressed as milliequivalents of active oxygen per kilogram of oil (meq O_2_/kg), represent the mean ± SD of triplicate determinations.

### 2.5. Quantification of PS and POPs

#### 2.5.1. Solid-Phase Extraction and Derivatization

A 120 mg oil sample was spiked with 2 μg 19-hydroxycholesterol and 20 μg cholestanol (internal standards), then dissolved in 5 mL *n*-hexane to prepare Sample S for PS and POPs extraction. An aminopropyl-silica SPE cartridge (ProElut NH_2_, 1.0 g/6 mL) was preconditioned with 1.0 g anhydrous sodium sulfate followed by equilibration with 2 × 5 mL *n*-hexane. After loading Sample S, matrix interferences (including triacylglycerols) were removed by washing with 2 × 5 mL *n*-hexane/diethyl ether (87:13, *v/v*). PS-containing Fraction A was eluted with 2 × 5 mL *n*-hexane/diethyl ether (40:60, *v/v*). Subsequently, POP-containing Fraction B was collected using 2 × 5 mL acetone. Both fractions underwent trimethylsilylation according to our established protocol [5]: 10 mL of each fraction was evaporated under nitrogen stream and derivatized with 100 μL MSHFBA/1-MIM (95:5, *v/v*) at 75 °C for 20 min.

#### 2.5.2. Phytosterol Analysis

Derivatized samples (100 μL) were diluted with 900 μL *n*-hexane prior to GC-MS analysis (Agilent 7890A GC/5973N MS, Agilent, Santa Clara, CA, USA). Separation was achieved using a DB-5MS capillary column (30 m × 0.25 mm ID × 0.25 μm phenyl arylene polymer film). The oven program was initiated at 100 °C (1 min hold), and then raised to 200 °C at a rate of 50 °C/min, to 250 °C at 20 °C/min, and finally to 300 °C at 1.5 °C/min, and held for 10 min. Helium carrier gas flowed at 1.2 mL/min. The injection volume was 1 μL in split mode (at a ratio of 10:1). Temperatures were maintained at 290 °C (injector), 300 °C (transfer line), and 230 °C (ion source). PS were identified in SIM mode (Table S1) through retention time and mass spectral matching against authentic standards (campesterol, stigmasterol, β-sitosterol). PS Quantitation employed internal standard calibration curves plotting (analyte peak area/cholestanol area) vs. (analyte concentration/cholestanol concentration).

#### 2.5.3. POPs Analysis

The derivatized POPs were identified and quantified using the procedure described by Hu et al. [29]. Chromatographic separation conditions for POPs were the same as those used for PS except the difference in injection mode. For POPs, the injection was in split-less mode. The SIM mode was used for identification and quantification of POPs, and the MS data and their chromatograms are shown in Table S1 and Figure S1. Quantitation of POPs utilized cholesterol oxidation products (COPs) calibration curves [17] due to structural similarity and POP standard unavailability. Eight-point COP curves (0.1–80 μg/g for 7α-OH, 7β-OH, 5,6α-epoxy, triol-cholesterol; 0.5–200 μg/g for 5,6β-epoxy and 7-keto-cholesterol) with 2 μg 19-hydroxycholesterol internal standard were constructed by plotting (analyte area/19-hydroxycholesterol area) vs. (analyte concentration/19-hydroxycholesterol concentration).

### 2.6. Statistical Analysis

Data processing utilized Microsoft Excel 365, OriginPro 2020, and IBM SPSS Statistics 25.0. Triplicate measurements are presented as mean ± SD. Significant differences (*p* < 0.05) were determined by one-way ANOVA with Tukey’s post hoc test.

## 3. Results and Discussion

### 3.1. Antioxidative Effects of Various Antioxidants on Camellia Seed Oil During Thermal Treatment

**Figure 1:**
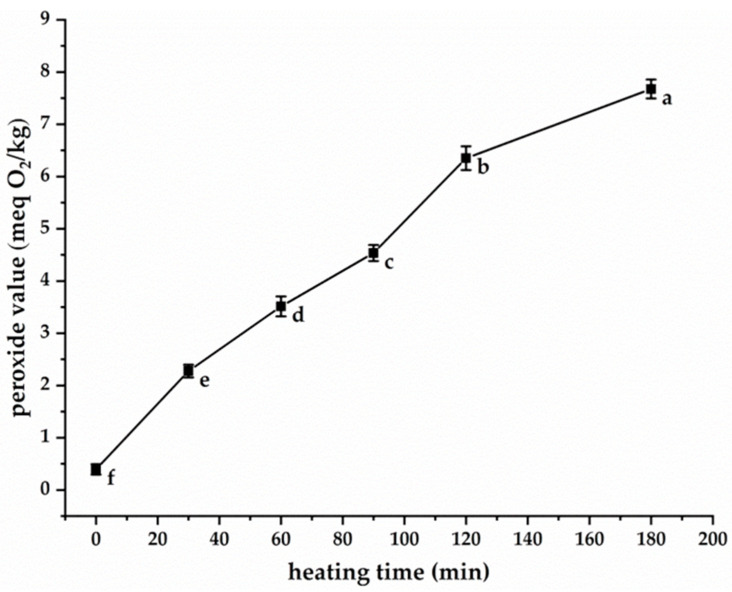
The change in PV of CSO heated at 180 °C. Different lowercase letters indicate significant differences (*p* < 0.05) in peroxide values among samples at different heating times.

PV serves as a reliable indicator for monitoring the oxidation progression of fats and oils. A principal advantage of PV quantification lies in its direct measurement of lipid peroxides, representing primary oxidation products. As demonstrated in Figure 1, the PV of purified CSO (without antioxidant addition) subjected to 180 °C heating exhibited a time-dependent increase from an initial value of 0.4 meq O_2_/kg to 7.67 meq O_2_/kg after 180 min thermal treatment. This observed increase can be attributed to the thermally induced oxidation of unsaturated constituents, notably PS and unsaturated fatty acids in CSO, with the following composition: 80.6% oleic acid, 7.8% linoleic acid, and 0.1% linolenic acid. Under elevated temperatures, these unsaturated molecules underwent selective hydrogen abstraction, generating various free radical species through homolytic cleavage mechanisms. These radicals subsequently react with atmospheric oxygen to form peroxyl radicals (LOO•), which function as chain carriers in the propagation phase of lipid oxidation. The chain reaction involves continuous hydrogen abstraction from adjacent lipid molecules and PS, potentially repeating thousands of times during thermal processing. This autocatalytic process leads to progressive accumulation of lipid hydroperoxides and PS hydroperoxides, thereby accounting for the PV increase observed in Figure 1. Notably, PS hydroperoxides exhibited particular instability, undergoing decomposition into multiple secondary oxidation products including 7α-OH-, 7β-OH-, 5,6β-epoxy-, 5,6α-epoxy-, 3β,5α,6β-triol-, and 7-keto-PS derivatives [14,17]. These secondary oxidation products have been associated with potential adverse health effects [30]. Consequently, developing effective strategies to inhibit or delay CSO oxidation, particularly PS degradation during thermal processing, represents a crucial research priority.

**Figure 2:**
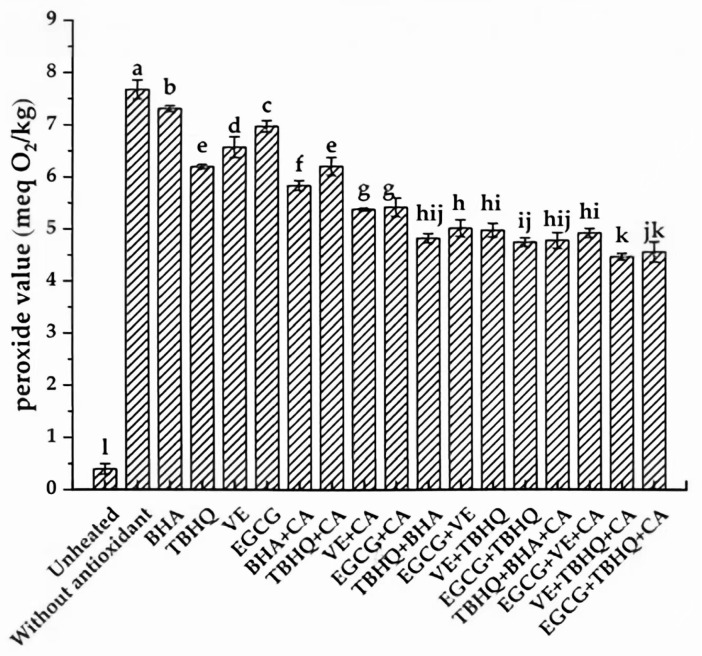
Influence of various antioxidants on PV of CSO heated at 180 °C. Different lowercase letters indicate significant differences (*p* < 0.05) in peroxide values between samples treated in different ways.

To mitigate the oxidation of CSO and PS, the application of antioxidants represents an effective, convenient, and economically viable strategy. Figure 2 illustrates the PVs of CSO subjected to heating at 180 °C for 3 h, both in the presence and absence of antioxidants. While the PV of the lipid matrix increased significantly after heating regardless of antioxidant addition, the PVs of CSO samples containing antioxidants were consistently lower than that of the blank control group (heated without antioxidants). This observation indicates the efficacy of antioxidants in retarding the oxidative degradation of CSO and PS. Among the single antioxidants tested, the antioxidant efficacy followed the descending order: TBHQ > VE > EGCG > BHA, with corresponding PVs of 6.20, 6.57, 6.96, and 7.31 meq O_2_/kg, respectively. The inclusion of CA as a synergist with single antioxidants further enhanced their antioxidative performance. This improvement can be attributed to CA’s ability to chelate metal ions, forming thermodynamically stable complexes and reducing their redox potentials, thereby diminishing their pro-oxidative effects. Additionally, CA facilitates the delocalization of unpaired electrons around the phenol rings of antioxidant radicals, forming stable resonance hybrids. These hybrids exhibit low reactivity and are less likely to initiate the formation of new radicals, effectively disrupting the chain reaction of free radical propagation [31]. Notably, the use of complex antioxidant systems, including binary mixed antioxidants and binary mixed antioxidants combined with CA, demonstrated superior efficacy in enhancing the oxidative stability of CSO and PS compared to single antioxidants or their combinations with synergists (Figure 2). The lowest PV (4.47 meq O_2_/kg) was observed in samples treated with a combination of VE, TBHQ, and CA. This synergistic effect likely arises from the complementary mechanisms of action exhibited by complex antioxidants, which may include enhanced free radical scavenging capabilities, suppression of oxidation initiators, and regeneration of primary antioxidants [22]. These findings highlight the potential of complex antioxidant systems in optimizing the oxidative stability of CSO and PS during thermal processing.

### 3.2. Effects of Antioxidants on PS Stability in Camellia Seed Oil Under Heating

Natural camellia seed oil contains both endogenous antioxidants (e.g., tocopherols, polyphenols, and squalene) and prooxidants (e.g., free fatty acids, peroxides, and chlorophyll) [32]. The coexistence of these substances interferes with the oxidation kinetics of triglycerides and phytosterols in the oil. To accurately evaluate the inhibitory effects of exogenous antioxidants on phytosterol oxidation, endogenous antioxidants and prooxidants should be removed; otherwise, their confounding effects would obscure the true efficacy of the tested antioxidants. Therefore, in this study, all endogenous antioxidants and prooxidants were systematically eliminated from the camellia seed oil prior to experimentation. This pretreatment ensures that the observed inhibition of sterol oxidation can be unequivocally attributed to the applied antioxidant. The impact of various antioxidants on the retention of PS in CSO subjected to heating at 180 °C for 3 h is presented in Table 1. The initial total PS content in unheated CSO was 4905.3 μg/g. Following thermal treatment, a reduction in PS content was observed across all samples. However, the extent of PS loss was significantly mitigated in samples supplemented with antioxidants, indicating that antioxidant addition effectively attenuates the thermal oxidation and degradation of PS. Among the tested samples, the combinations of VE + TBHQ + CA and EGCG + TBHQ + CA exhibited the highest PS retention, with remaining PS contents of 3921.4 μg/g and 3883.7 μg/g, respectively. Based on the initial PS content, the corresponding PS loss rates were calculated to be 20.1% and 20.8%. In contrast, the highest PS loss was observed in the sample containing BHA, with a remaining PS content of 3452.6 μg/g. At the individual PS level, the lowest degradation rates were similarly observed in samples treated with VE + TBHQ + CA and EGCG + TBHQ + CA, whereas the highest degradation occurred in the BHA-supplemented sample. These findings suggest that synthetic antioxidants, such as BHA, do not consistently exhibit superior efficacy in inhibiting PS oxidation and degradation. This phenomenon may be attributed to the following factors: (1) Complex antioxidant systems demonstrate synergistic interactions, enhancing their ability to suppress PS oxidation more effectively than single antioxidants [33]; (2) BHA is prone to thermal decomposition at 180 °C, resulting in diminished antioxidant activity and consequently greater PS loss [33,34]. These results underscore the importance of optimizing antioxidant formulations to maximize the thermal stability of PS in CSO during high-temperature processing.

### 3.3. Influence of Various Antioxidants on POPs Content in CSO

#### 3.3.1. Influence of Various Antioxidants on POPs Formation

Under thermal treatment at 180 °C, POPs were generated progressively alongside the degradation of PS. The effects of various antioxidants on POP formation in CSO are summarized in Table 2. Compared to the blank control, antioxidant supplementation significantly inhibited POP generation. Specifically, the generation of POPs was reduced by 222.1, 336.2, 288.5, and 274.3 μg/g in samples treated with BHA, TBHQ, VE, and EGCG, respectively, relative to the blank group. Similarly, Tabee et al. [35] demonstrated that the addition of VE can significantly (*p* < 0.05) inhibit the formation of POPs in refined olive oil after 6, 9, and 12 h of heating at 180 °C. Notably, combined antioxidant systems—including single antioxidants with CA, binary mixtures, and ternary mixtures with CA—exhibited superior inhibition efficacy compared to individual antioxidants. Among these formulations, the VE + TBHQ + CA and EGCG + TBHQ + CA combinations demonstrated the strongest inhibitory effects, whereas BHA alone showed the lowest efficacy. After 3 h of heating, POP levels in these groups were 518.1 μg/g, 569.8 μg/g, and 882.3 μg/g, respectively. These results underscore the enhanced performance of combined antioxidant systems in suppressing POP generation, likely attributable to synergistic mechanisms [33,36] including: (1) Enhanced scavenging of carbon-centered radicals (C•), peroxyl radicals (LOO•), and alkoxyl radicals (LO•); (2) Inhibition of oxidation initiators (e.g., transition metals and hydroxyl radicals, HO•); (3) Regeneration of primary antioxidants. Collectively, the combined antioxidant systems improve PS oxidative stability more effectively than single antioxidants.

The formation of POPs and their final concentration in an oil matrix may be affected by many factors, such as heating temperature and time, dissolved oxygen, triglyceride profiles, and the presence or absence of antioxidants. In a study reported by Tabee et al. [35], high-oleic rapeseed oil and refined olive oil—both with triglyceride profiles similar to the CSO used in our study—were heated at 180 °C for 3 h. Despite these comparable conditions, the POP contents in both oils (which lacked exogenous antioxidants) differed significantly from our results. Their study reported total POP concentrations of 66.3 μg/g and 14.9 μg/g for high-oleic rapeseed oil and refined olive oil, respectively. In contrast, our study observed a total POP concentration of 1104.4 μg/g in CSO under identical heating conditions. This disparity can be attributed to two critical factors: (1) PS concentration: The CSO in our study was artificially enriched with PS (500 mg/100 g oil), whereas the oils in Tabee’s study contained native PS levels (e.g., total sterols in refined olive oil: 137.3 mg/100 g oil). Higher PS concentrations in our system provided more oxidation substrates, accelerating POP formation. (2) Endogenous antioxidants: The oils in Tabee’s study contained natural tocopherols (e.g., high-oleic rapeseed oil: 63.2 mg/100 g; refined olive oil: 15.7 mg/100 g), which scavenge radicals and inhibit PS oxidation. Conversely, our PS-enriched CSO lacked these native antioxidants, increasing its susceptibility to thermal oxidation and accelerating POP formation.

#### 3.3.2. Inhibitory Effects of Various Antioxidants on the C7 Hydroxy/Ketone Pathway in PS Oxidation

As demonstrated in our study, campesterol, stigmasterol, and β-sitosterol displayed analogous degradation profiles, which can be rationalized by their comparable dissociation enthalpies at the C5 and C6 double bonds within the steroid nucleus B-ring [37]. Therefore, the total concentrations of individual POPs derived from the three PS were summed to investigate the inhibitory effects of antioxidants on PS oxidation pathways. Thermal treatment of PS induces a free radical-mediated oxidation process, wherein POP formation proceeds via two distinct pathways [36,38]: (1) the C7 hydroxy/ketone pathway and (2) the C5/C6 epoxy pathway. In the C7 hydroxy/ketone pathway, the predominant POPs generated were 7α-hydroxy-, 7β-hydroxy-, and 7-keto-PS. Mechanistically, PS oxidation is initiated by HO• or transition metal ions, leading to the formation of a carbon-centered radical at C7 [PS(C7)•]. Subsequent reaction of PS(C7)• with molecular oxygen (O_2_) yields a PS-peroxyl radical [PS(C7)-OO•]. This peroxyl radical abstracts a hydrogen atom from adjacent native PS molecules, producing PS hydroperoxide [PS(C7)-OOH] and regenerating a new PS(C7)• radical, thereby propagating the chain reaction. The hydroperoxide [PS(C7)-OOH] undergoes transition metal-catalyzed homolytic cleavage or thermolysis to form an alkoxyl radical [PS(C7)-O•]. Subsequently, [PS(C7)-O•] abstracts a hydrogen atom from the C7 position of native PS, yielding 7β/α-hydroxy-PS. Further oxidation of 7β/α-hydroxy-PS culminates in the formation of 7-keto-PS. Thus, effective inhibition of the C7 hydroxy/ketone pathway requires either suppression of chain initiation or interruption of propagation steps.

**Figure 3:**
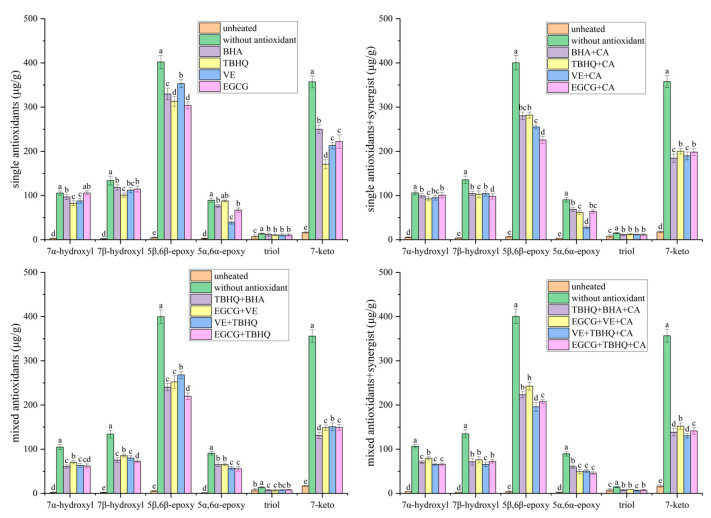
Influence of different antioxidants on main POPs in heated oil. Different lowercase letters indicate significant differences (*p* < 0.05) between samples treated in different ways.

Quantitative analysis revealed that the antioxidant systems exhibited a hierarchical inhibitory efficacy on the formation of 7β/α-hydroxy- and 7-keto-PS, ranked as follows: binary mixed antioxidants + CA > binary mixed antioxidants > single antioxidant + CA > single antioxidant. Notably, the ternary combination of VE, TBHQ, and CA achieved a PS degradation inhibition rate of 42% (Table 1). Concurrently, this formulation reduced the formation of 7β-hydroxy-PS, 7α-hydroxy-PS, and 7-keto-PS by 50%, 52%, and 56%, respectively, relative to the blank control group after heating at 180 °C for 3 h (Figure 3). In contrast, treatment with BHA alone resulted in only a 14% inhibition of PS degradation (Table 1), with reductions of 22%, 23%, and 19% in 7β-hydroxy-, 7α-hydroxy-, and 7-keto-PS levels, respectively (Figure 3). The superior performance of the VE + TBHQ + CA system can be attributed to two synergistic mechanisms: (1) The combined action of VE and TBHQ enhances scavenging of HO• radicals, while CA chelates transition metal ions to form thermodynamically stable complexes, thereby lowering their redox activity and delaying oxidation initiation; (2) The synergy between VE and TBHQ improves hydrogen donation efficiency to quench radicals such as PS(C7)•, PS(C7)-OO•, and PS(C7)-O•, effectively terminating chain propagation and suppressing 7β/α-hydroxy-PS formation. Additionally, the diminished conversion of 7β/α-hydroxy-PS to 7-keto-PS reflects the inhibition of secondary oxidation steps.

#### 3.3.3. Inhibitory Effects of Various Antioxidants on the C5/C6 Epoxy Pathway in PS Oxidation

The C5/C6 epoxy pathway constitutes an additional oxidative route for PS, with the primary POPs being 5α,6α-epoxy-PS, 5β,6β-epoxy-PS, and triol-PS. Notably, this pathway proceeds via a mechanism distinct from hydrogen abstraction, wherein peroxyl radicals directly add to the C5 = C6 double bond of PS. Specifically, hydroperoxyl radicals (e.g., PS(C7)-OO• or lipid-derived LOO•) react with the C5 = C6 double bond, leading to the formation of α,β-epoxide-PS through a process accompanied by alkoxyl radical release [36]. Alternatively, PS epoxides may arise from the interaction of lipid hydroperoxides with PS [38]. Triol-PS, a transformation product derived from epoxide-PS, was detected at trace levels compared with 5α,6α-epoxy-PS and 5β,6β-epoxy-PS (Figure 3). Consequently, this study focused on the predominant 5α,6α-epoxy-PS and 5β,6β-epoxy-PS to evaluate the efficacy of various antioxidants in suppressing the C5/C6 epoxy pathway.

Similarly to the inhibition observed in the C7 hydroxy/ketone pathway, the antioxidant systems exhibited a hierarchy in suppressing 5α,6α-epoxy- and 5β,6β-epoxy-PS formation, ranked as follows: binary mixed antioxidants + CA > binary mixed antioxidants > single antioxidant + CA > single antioxidant. Notably, the ternary combination of VE, TBHQ, and CA reduced 5α,6α-epoxy- and 5β,6β-epoxy-PS levels by 50% and 56%, respectively, relative to the blank control (Figure 3). In contrast, treatment with BHA alone achieved only 22% and 19% reductions in 5α,6α-epoxy- and 5β,6β-epoxy-PS, respectively (Figure 3). The superior performance of the VE + TBHQ + CA system is mechanistically linked to two synergistic actions: (1) The cooperative interaction of VE and TBHQ enhances hydrogen donation capacity, effectively quenching hydroperoxyl radicals (e.g., PS(C7)-OO• and LOO•) and thereby diminishing their reactivity toward the C5 = C6 double bond; (2) Simultaneously, CA chelates transition metal ions to form redox-inert complexes, suppressing metal-catalyzed epoxidation and terminating radical chain propagation. These combined effects collectively attenuate PS epoxide formation and stabilize the oxidative cascade.

## 4. Conclusions

In conclusion, this study comprehensively evaluated the oxidative stability of PS in CSO during high-temperature heating (180 °C) and systematically investigated the efficacy of various antioxidant strategies in mitigating PS degradation and POP formation. The experimental results demonstrated that binary mixed antioxidants + CA had the best antioxidant effect, followed by binary mixed antioxidants, then single antioxidant + CA, with single antioxidant alone showing the lowest efficacy. Notably, the combination of VE, TBHQ, and CA exhibited the most pronounced protective effect, achieving a 42% inhibition rate of PS degradation and reducing total POPs by 50–56% compared to the control. Mechanistic evaluation demonstrated that the ternary antioxidant system (VE + TBHQ + CA) attenuated the formation of POPs through two distinct pathways: (1) In the C7 oxidation pathway, the system reduced 7β-hydroxy-, 7α-hydroxy-, and 7-keto-PS by 50%, 52%, and 56%, respectively, primarily by scavenging initiators and chelating metals, and quenching propagation radicals; (2) Concurrently, in the C5/C6 epoxy pathway, it inhibited the formation of 5α,6α-epoxy- and 5β,6β-epoxy-PS by 50% and 56%, mainly by scavenging hydroperoxyl radicals and inhibiting metal-catalyzed epoxidation. In contrast, single antioxidants such as BHA exhibited significantly lower efficacy, inhibiting PS degradation by only 14%. The superior performance of mixed antioxidants (e.g., VE + TBHQ + CA) was attributed to their synergistic mechanisms: VE acted as a hydrogen donor, TBHQ efficiently quenched peroxyl radicals, and CA chelated pro-oxidant metal ions, collectively disrupting both initiation and propagation stages of PS oxidation. These findings not only elucidate the oxidation pathways of PS in CSO but also provide a scientifically validated, practical solution for enhancing PS thermal stability in edible oil. The optimized antioxidant protocol holds significant potential for industrial applications, particularly in high-temperature cooking processes, to minimize POP generation and ensure the nutritional quality and safety of camellia seed oil.

## Figures and Tables

**Table 1:** Impact of Antioxidant Addition on PS Retention (μg/g) in CSO Under Heating at 180 °C for 3 h.

Antioxidants	Campesterol	Stigmasterol	β-sitosterol	Total PS ^※^
Heated purified oil	ND ^※※^	ND	ND	ND
Unheated PS-enriched oil	769.7 ± 8.3 ^a^	848.1 ± 17.1 ^a^	3293.4 ± 73.0 ^a^	4905.3 ± 73.5 ^a^
Without antioxidant	520.0 ± 10.1 ^l^	585.6 ± 9.8 ^j^	2122.1 ± 46.2 ^k^	3220.1 ± 35.7 ^n^
BHA	572.3 ± 7.8 ^k^	615.3 ± 9.8 ^i^	2270.5 ± 56.9 ^j^	3452.6 ± 51.0 ^m^
TBHQ	600.6 ± 7.0 ^ij^	637.4 ± 12.4 ^gh^	2363.2 ± 59.5 ^hi^	3596.5 ± 57.9 ^jk^
VE	588.1 ± 15.5 ^ijk^	634.2 ± 11.1 ^hi^	2317.6 ± 53.1 ^ij^	3529.1 ± 33.1 ^kl^
EGCG	584.7 ± 8.7 ^jk^	629.2 ± 16.9 ^hi^	2309.3 ± 47.5 ^ij^	3517.3 ± 47.1 ^lm^
BHA + CA	603.8 ± 11.9 ^hi^	649.3 ± 12.4 ^fgh^	2378.7 ± 51.6 f^ghi^	3622.7 ± 40.2 ^hij^
TBHQ + CA	604.1 ± 15.2 ^ghi^	647.8 ± 16.3 ^fgh^	2370.4 ± 46.5 ^ghi^	3611.2 ± 32.3 ^ij^
VE + CA	620.7 ± 8.1 ^fgh^	663.4 ± 9.3 ^ef^	2414.7 ± 67.1 ^efgh^	3692.4 ± 60.1 ^fgh^
EGCG + CA	621.6 ± 13.7 ^fg^	658.3 ± 9.9 ^efg^	2401.1 ± 71.7 ^efgh^	3671.5 ± 54.2 ^ghi^
TBHQ + BHA	643.7 ± 17.7 ^bcde^	679.2 ± 19.3 ^cde^	2499.5 ± 61.5 ^bcd^	3810.2 ± 45.4 ^de^
EGCG + VE	626.3 ± 11.4 ^ef^	666.8 ± 11.7 ^def^	2457.6 ± 43.4 ^def^	3742.2 ± 32.3 ^efg^
VE + TBHQ	633.7 ± 11.8 ^def^	671.8 ± 16.0 ^cde^	2452.1 ± 56.7 ^defg^	3749.7 ± 49.1 ^ef^
EGCG + TBHQ	649.9 ± 13.7 ^bcd^	689.8 ± 14.9 ^bc^	2509.8 ± 49.6 ^bcd^	3840.8 ± 37.1 ^cd^
TBHQ + BHA + CA	648.3 ± 9.4 ^bcd^	687.1 ± 18.5 ^bcd^	2504.2 ± 63.2 ^bcd^	3833.1 ± 62.8 ^cd^
EGCG + VE + CA	641.9 ± 10.4 ^cde^	676.5 ± 13.7 ^cde^	2464.5 ± 58.7 ^cde^	3775.3 ± 51.6 ^de^
VE + TBHQ + CA	660.8 ± 15.9 ^b^	701.7 ± 16.2 ^b^	2570.1 ± 58.3 ^b^	3921.4 ± 42.7 ^b^
EGCG + TBHQ + CA	658.4 ± 12.5 ^bc^	691.3 ± 15.7 ^bc^	2542.7 ± 52.4 ^bc^	3883.7 ± 43.0 ^bc^

^※^ Total phytosterols represent the sum of campesterol, stigmasterol, and β-sitosterol. The data shown in the table are presented as mean ± standard deviation (*n* = 3). Different lowercase letters within the same column indicate significant differences (*p* < 0.05) between samples. ^※※^ ND were below the detection limit.

**Table 2:** Influence of different antioxidants on contents of POPs (μg/g) in CSO heated at 180 °C.

Antioxidants	Campesterol-POPs	Stigmasterol-POPs	β-sitosterol-POPs	Total POPs ^※^
Heated purified oil	ND ^※※^	ND	ND	ND
Unheated PS-enriched oil	11.9 ± 1.3 ^j^	7.5 ± 0.7 ^m^	20.1 ± 1.4 ^i^	39.5 ± 3.5 ^j^
Without antioxidant	268.9 ± 13.1 ^a^	172.2 ± 8.2 ^a^	663.3 ± 16.5 ^a^	1104.4 ± 37.8 ^a^
BHA	216.7 ± 11.7 ^b^	143.3 ± 8.8 ^b^	522.3 ± 11.6 ^b^	882.3 ± 32.1 ^b^
TBHQ	189.0 ± 7.9 ^d^	128.6 ± 6.1 ^cd^	450.6 ± 19.8 ^d^	768.2 ± 34.0 ^d^
VE	201.1 ± 7.2 ^c^	129.0 ± 5.8 ^c^	485.8 ± 14.7 ^c^	815.9 ± 27.9 ^c^
EGCG	204.3 ± 9.4 ^c^	133.0 ± 6.7 ^c^	492.8 ± 18.1 ^c^	830.1 ± 34.3 ^c^
BHA + CA	185.6 ± 6.3 ^d^	120.8 ± 5.1 ^de^	444.3 ± 12.6 ^d^	750.7 ± 24.1 ^d^
TBHQ + CA	186.0 ± 9.0 ^d^	119.6 ± 5.5 ^e^	438.2 ± 13.4 ^d^	743.8 ± 27.9 ^d^
VE + CA	168.4 ± 5.5 ^e^	111.5 ± 4.0 ^f^	416.1 ± 21.9 ^e^	696.0 ± 31.5 ^e^
EGCG + CA	169.6 ± 8.3 ^e^	107.0 ± 4.8 ^fg^	403.6 ± 17.9 ^e^	680.2 ± 31.2 ^e^
TBHQ + BHA	148.7 ± 6.3 ^gh^	96.5 ± 3.8 ^ijk^	366.8 ± 13.6 ^f^	612.0 ± 23.7 ^fg^
EGCG + VE	164.0 ± 6.3 ^ef^	104.8 ± 3.3 ^fgh^	364.5 ± 13.5 ^f^	633.3 ± 23.2 ^f^
VE + TBHQ	156.7 ± 5.8 ^fg^	100.6 ± 5.0 ^ghi^	372.4 ± 17.9 ^f^	629.7 ± 28.7 ^f^
EGCG + TBHQ	135.5 ± 5.4 ^i^	90.5 ± 4.1 ^jkl^	318.3 ± 10.0 ^gh^	544.3 ± 19.6 ^hi^
TBHQ + BHA + CA	142.6 ± 7.1 ^hi^	91.2 ± 5.0 ^jk^	338.9 ± 10.5 ^g^	572.7 ± 22.6 ^gh^
EGCG + VE + CA	147.1 ± 5.5 ^gh^	97.9 ± 6.6 ^hij^	337.5 ± 16.6 ^g^	582.5 ± 28.8 ^gh^
VE + TBHQ + CA	134.1 ± 8.3 ^i^	83.2 ± 4.2 ^l^	300.8 ± 10.9 ^h^	518.1 ± 23.5 ^i^
EGCG + TBHQ + CA	141.1 ± 6.2 ^hi^	89.0 ± 3.7 ^kl^	339.7 ± 10.9 ^g^	569.8 ± 20.9 ^h^

^※^ Total POPs represents the sum of campesterol-POPs, stigmasterol-POPs, and β-sitosterol-POPs. The data shown in the table was expressed as mean ± standard deviation (*n* = 3), and different letters within the same column indicate significant differences (*p* < 0.05) between samples. ^※※^ ND were below the detection limit.

## Data Availability

The original contributions presented in this study are included in the article; further inquiries can be directed to the corresponding author.

## Supplementary Materials

The following supporting information can be downloaded at: https://www.mdpi.com/article/10.3390/foods14132297/s1, Figure S1: Chromatograms of TMS derivatives of phytosterols and phytosterol oxidation products analyzed by GC-MS. Table S1. MS data and retention times (RTs) for phytosterols and phytosterol oxidation products analyzed by GC-MS.

## References

[1] Yang K.-M., Hsu F.-L., Chen C.-W., Hsu C.-L., Cheng M.-C. (2018). Quality characterization and oxidative stability of camellia seed oils produced with different roasting temperatures. J. Oleo Sci..

[2] Zhang S., Pan Y.G., Zheng L., Yang Y., Zheng X., Ai B., Xu Z., Sheng Z. (2019). Application of steam explosion in oil extraction of camellia seed (*Camellia oleifera* Abel.) and evaluation of its physicochemical properties, fatty acid, and antioxidant activities. Food Sci. Nutr..

[3] Cao J., Jiang X., Chen Q., Zhang H., Sun H., Zhang W.-M., Li C. (2020). Oxidative stabilities of olive and camellia oils: Possible mechanism of aldehydes formation in oleic acid triglyceride at high temperature. LWT Food Sci. Technol..

[4] Moreau R.A., Nyström L., Whitaker B.D., Winkler-Moser J.K., Baer D.J., Gebauer S.K., Hicks K.B. (2018). Phytosterols and their derivatives: Structural diversity, distribution, metabolism, analysis, and health-promoting uses. Prog. Lipid Res..

[5] Xu B., You S., Zhou L., Kang H., Luo D., Ma H., Han S. (2020). Simultaneous determination of free phytosterols and tocopherols in vegetable oils by an improved SPE–GC–FID method. Food Anal. Methods.

[6] Gylling H., Miettinen T.A. (2005). The effect of plant stanol-and sterol-enriched foods on lipid metabolism, serum lipids and coronary heart disease. Ann. Clin. Biochem..

[7] Plat J., Mensink R.P. (2005). Plant stanol and sterol esters in the control of blood cholesterol levels: Mechanism and safety aspects. Am. J. Cardiol..

[8] Wong A. (2014). Chemical and microbiological considerations of phytosterols and their relative efficacies in functional foods for the lowering of serum cholesterol levels in humans: A review. J. Funct. Foods.

[9] Marangoni F., Poli A. (2010). Phytosterols and cardiovascular health. Pharmacol. Res..

[10] Awad A., Downie A., Fink C., Kim U. (2000). Dietary phytosterol inhibits the growth and metastasis of MDA-MB-231 human breast cancer cells grown in SCID mice. Anticancer Res..

[11] Suttiarporn P., Chumpolsri W., Mahatheeranont S., Luangkamin S., Teepsawang S., Leardkamolkarn V. (2015). Structures of phytosterols and triterpenoids with potential anti-cancer activity in bran of black non-glutinous rice. Nutrients.

[12] Lin Y., Knol D., Menéndez-Carreño M., Blom W.A., Matthee J., Janssen H.G., Trautwein E.A. (2016). Formation of Plant Sterol Oxidation Products in Foods during Baking and Cooking Using Margarine without and with Added Plant Sterol Esters. J. Agric. Food Chem..

[13] Lin Y., Knol D., Valk I., van Andel V., Friedrichs S., Lütjohann D., Hrncirik K., Trautwein E.A. (2017). Thermal stability of plant sterols and formation of their oxidation products in vegetable oils and margarines upon controlled heating. Chem. Phys. Lipids.

[14] Xu B., You S., Zhang L., Ma F., Zhang Q., Luo D., Li P. (2022). Comparative analysis of free/combined phytosterols--degradation and differential formation of oxidation products during heating of sunflower seed oil. LWT Food Sci. Technol..

[15] Lin Y., Knol D., Trautwein E.A. (2016). Phytosterol oxidation products (POP) in foods with added phytosterols and estimation of their daily intake: A literature review. Eur. J. Lipid Sci. Technol..

[16] Wang M., Lu B. (2018). How do oxyphytosterols affect human health?. Trends Food Sci. Technol..

[17] Zhao Y., Yang B., Xu T., Wang M., Lu B. (2019). Photooxidation of phytosterols in oil matrix: Effects of the light, photosensitizers and unsaturation degree of the lipids. Food Chem..

[18] Xu G., Sun J., Liang Y., Yang C., Chen Z.-Y. (2011). Interaction of fatty acids with oxidation of cholesterol and β-sitosterol. Food Chem..

[19] Hu P.C., Chen B.H. (2002). Effects of riboflavin and fatty acid methyl esters on cholesterol oxidation during illumination. J. Agric. Food Chem..

[20] Ma J., Ye H., Rui Y., Chen G., Zhang N. (2011). Fatty acid composition of *Camellia oleifera* oil. J. Verbraucherschutz Leb..

[21] Barriuso B., Ansorena D., Poyato C., Astiasarán I. (2015). Cholesterol and stigmasterol within a sunflower oil matrix: Thermal degradation and oxysterols formation. Steroids.

[22] Kmiecik D., Korczak J., Rudzińska M., Gramza-Michałowska A., Hęś M., Kobus-Cisowska J. (2015). Stabilisation of phytosterols by natural and synthetic antioxidants in high temperature conditions. Food Chem..

[23] Zhang Y., Shen Y., Zhu Y., Xu Z. (2015). Assessment of the correlations between reducing power, scavenging DPPH activity and anti-lipid-oxidation capability of phenolic antioxidants. LWT Food Sci. Technol..

[24] Hu Y., Huang W., Li M., Wang M., Zhao Y., Xu T., Zhang L., Lu B., He Y. (2017). Metal ions accelerated phytosterol thermal degradation on Ring A & Ring B of steroid nucleus in oils. Food Res. Int..

[25] Xu B., Zhang L., Wang H., Luo D., Li P. (2014). Characterization and authentication of four important edible oils using free phytosterol profiles established by GC-GC-TOF/MS. Anal. Methods.

[26] Rokosik E., Dwiecki K., Rudzińska M., Siger A., Polewski K. (2019). Column chromatography as a method for minor components removal from rapeseed oil. Grasas Aceites.

[27] Abad A., Shahidi F. (2020). A robust stripping method for the removal of minor components from edible oils. Food Prod. Process. Nutr..

[28] Shantha N.C., Decker E.A. (1994). Rapid, sensitive, iron-based spectrophotometric methods for determination of peroxide values of food lipids. J. AOAC Int..

[29] Hu Y., Yang G., Huang W., Lai S., Ren Y., Huang B., Zhang L., Li P., Lu B. (2015). Development and validation of a gas chromatography-mass spectrometry method for determination of sterol oxidation products in edible oils. RSC Adv..

[30] Alemany L., Barbera R., Alegría A., Laparra J. (2014). Plant sterols from foods in inflammation and risk of cardiovascular disease: A real threat?. Food Chem. Toxicol..

[31] Shahidi F., Zhong Y. (2010). Lipid oxidation and improving the oxidative stability. Chem. Soc. Rev..

[32] Jing Y., Heqin Y., Yougen W., Yong W., Pengguo X. (2022). Quality Evaluation of the Oil of *Camellia* spp.. Foods.

[33] Liu X., Zheng Z., Liu Y. (2025). Lipophilic antioxidants in edible oils: Mechanisms, applications and interactions. Food Res. Int..

[34] Kmiecik D., Korczak J., Rudzińska M., Michałowska A.G., Hęś M. (2009). Stabilization of phytosterols in rapeseed oil by natural antioxidants during heating. Eur. J. Lipid Sci. Technol..

[35] Tabee E., Azadmard-Damirchi S., Jägerstad M. (2008). Effects of α-Tocopherol on Oxidative Stability and Phytosterol Oxidation During Heating in Some Regular and High-Oleic Vegetable Oils. J. Am. Oil Chem. Soc..

[36] Iuliano L. (2011). Pathways of cholesterol oxidation via non-enzymatic mechanisms. Chem. Phys. Lipids.

[37] Yang B.-W., Lu B.-Y., Zhao Y.-J., Luo J.-Y., Hong X. (2020). Formation of phytosterol photooxidation products: A chemical reaction mechanism for light-induced oxidation. Food Chem..

[38] Spiteller G. (2006). Peroxyl radicals: Inductors of neurodegenerative and other inflammatory diseases. Their origin and how they transform cholesterol, phospholipids, plasmalogens, polyunsaturated fatty acids, sugars, and proteins into deleterious products. Free Radic. Biol. Med..

