# Supplementary material for: Oxidative Stability of Phytosterols in Camellia Seed Oil During Heating: The Impact of Different Antioxidants
Source: Foods. 2025 Jun 28;14(13):2297. doi: 10.3390/foods14132297 (PMC12249176; doi:10.3390/foods14132297)
Supplement: Supplementary file 1 [file foods-14-02297-s001.zip › foods-3711208-supplementary.pdf]

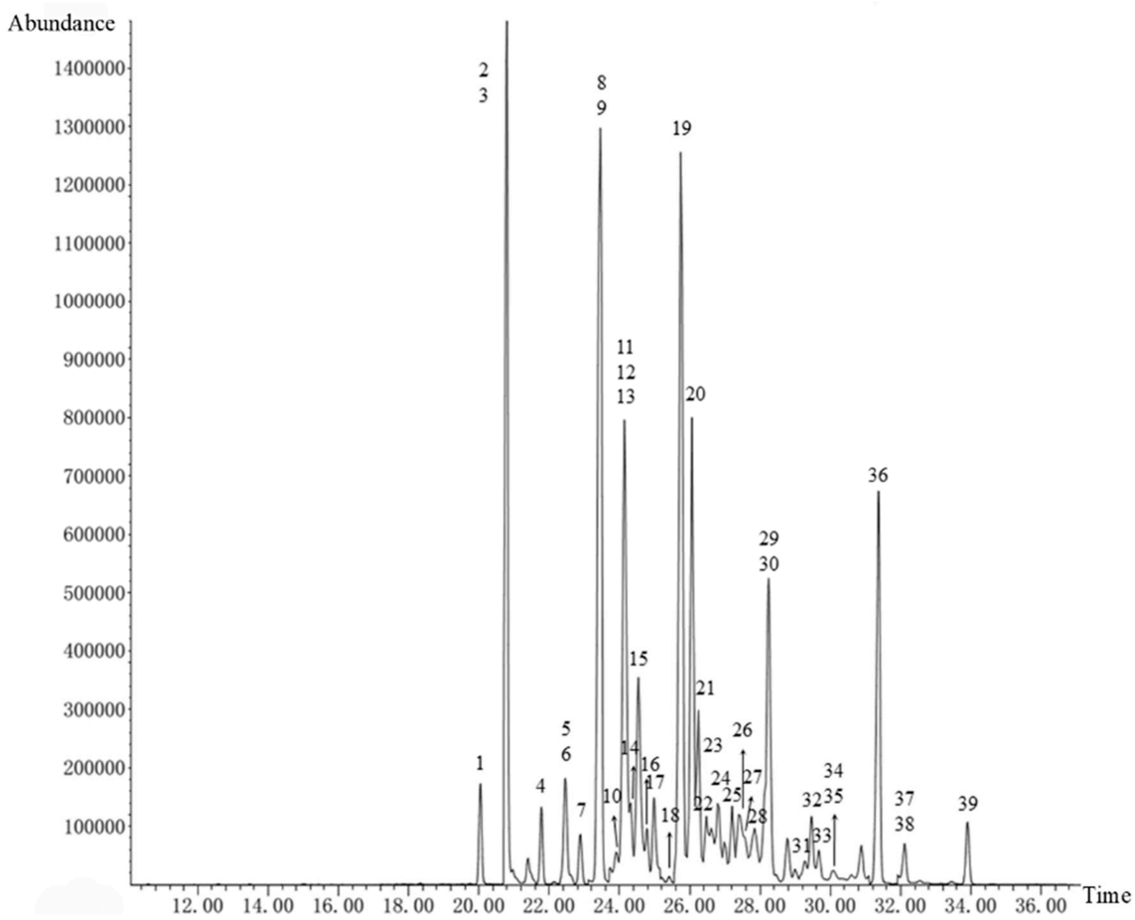

Figure S1. Chromatograms of TMS derivatives of phytosterols and phytosterol oxidation products analyzed by GC-MS.

Chromatographic peak labeling:

1. 7α-Hydroxycholesterol; 2. Cholesterol; 3. 7α-Hydroxybrassicasterol; 4. Brassicasterol; 5. 7α-Hydroxycampesterol; 6. 6β-Hydroxybrassicasterol

7. 7α-Hydroxystigmasterol; 8. 7β-Hydroxycholesterol; 9. Campesterol; 10. 5β,6β-Epoxycholesterol; 11. 7β-Hydroxybrassicasterol; 12. Stigmasterol

13. 6β-Hydroxycampesterol; 14. 5α,6α-Epoxycholesterol; 15. 7α-Hydroxysitosterol; 16. 6β-Hydroxystigmasterol; 17. 5β,6β-Epoxybrassicasterol;

18. 5α,6α-Epoxybrassicasterol; 19. β-Sitosterol; 20. 7β-Hydroxycampesterol; 21. 7β-Hydroxystigmasterol; 22. 6β-Hydroxysitosterol; 23. Trihydroxycholesterol;

24. 5β,6β-Epoxycampesterol; 25. 5α,6α-Epoxycampesterol; 26. Trihydroxybrassicasterol; 27. 5β,6β-Epoxystigmasterol; 28. 5α,6α-Epoxystigmasterol;

29. 7-Ketocholesterol; 30. 7β-Hydroxysitosterol; 31. 5β,6β-Epoxysitosterol; 32. 7-Ketobrassicasterol; 33. Trihydroxycampesterol; 34. 5α,6α-Epoxysitosterol;

35. Trihydroxystigmasterol; 36. 7-Ketocampesterol; 37. Trihydroxysitosterol; 38. 7-Ketostigmasterol; 39. 7-Ketositosterol

Table S1. MS data and retention times (RTs) for phytosterols and phytosterol oxidation products analyzed by GC-MS.

No.	Compound	RTs (min)	Quantitative ion (*m*/*z*)	Qualitative ions (*m*/*z*)		
1	7α-Hydroxycholesterol	20.030	456	546	531	441
2	Cholesterol	20.787	485	368	329	353
3	7α-Hydroxybrassicasterol	20.829	468	558	543	453
4	Brassicasterol	21.777	470	380	341	365
5	7α-Hydroxycampesterol	22.438	470	560	545	455
6	6β-Hydroxybrassicasterol	22.479	415	468	453	543
7	7α-Hydroxystigmasterol	22.880	482	572	557	467
8	7β-Hydroxycholesterol	23.387	456	546	531	441
9	Campesterol	23.465	472	382	343	367
10	5β,6β-Epoxycholesterol	23.926	474	384	459	445
11	7β-Hydroxybrassicasterol	24.123	468	558	543	453
12	Stigmasterol	24.133	484	394	355	379
13	6β-Hydroxycampesterol	24.179	417	470	455	545
14	5α,6α-Epoxycholesterol	24.293	474	384	459	445
15	7α-Hydroxysitosterol	24.548	484	574	559	469
16	6β-Hydroxystigmasterol	24.840	429	482	467	557
17	5β,6β-Epoxybrassicasterol	25.049	486	396	471	457
18	5α,6α-Epoxybrassicasterol	25.409	486	396	471	457
19	β-Sitosterol	25.770	486	396	357	381
20	7β-Hydroxycampesterol	26.052	470	560	545	455
21	7β-Hydroxystigmasterol	26.234	482	572	557	467
22	6β-Hydroxysitosterol	26.463	431	484	469	559
23	Trihydroxycholesterol	26.643	403	456	546	441
24	5β,6β-Epoxycampesterol	26.835	488	398	473	459
25	5α,6α-Epoxycampesterol	27.219	488	398	473	459
26	Trihydroxybrassicasterol	27.385	415	468	558	453
27	5β,6β-Epoxystigmasterol	27.544	500	410	485	471
28	5α,6α-Epoxystigmasterol	27.918	500	410	485	471
29	7-Ketocholesterol	28.224	472	382	367	457
30	7β-Hydroxysitosterol	28.258	484	574	559	469
31	5β,6β-Epoxysitosterol	29.290	502	412	487	473
32	7-Ketobrassicasterol	29.466	484	394	379	469
33	Trihydroxycampesterol	29.566	417	470	560	455
34	5α,6α-Epoxysitosterol	29.685	502	412	487	473
35	Trihydroxystigmasterol	30.251	572	482	429	467
36	7-Ketocampesterol	31.380	486	396	381	471
37	Trihydroxysitosterol	32.067	484	431	574	469
38	7-Ketostigmasterol	32.126	498	408	393	483
39	7-Ketositosterol	33.906	500	410	395	485
